# BCG Vaccination and Tuberculosis in Japan

**DOI:** 10.2188/jea.13.127

**Published:** 2007-11-30

**Authors:** Mahbubur Rahman, Osamu Takahashi, Masashi Goto, Tsuguya Fukui

**Affiliations:** 1 Department of General Medicine and Clinical Epidemiology, Kyoto University Graduate School of Medicine.

**Keywords:** BCG, cost-effectiveness, tuberculosis, Japan

## Abstract

This paper summarizes *Bacillus Calmette-Guérin* (BCG) vaccination and revaccination policies in Japan, its cost-effectiveness, side effects, proposed selective vaccination strategy, and present tuberculosis situation in Japanese perspectives based on Medline database and other published reports. Universal BCG vaccination in infants and revaccination among children were not found economically justifiable. Overall tuberculosis incidence in Japan is higher than that of other developed countries. Trend of decline in tuberculosis incidence is similar to that of the countries where universal BCG vaccination has never been implemented. In the recent years, the number of tuberculosis group infection has been escalating. Since BCG revaccination program has already been discontinued, a consensus on universal BCG vaccination is also essential based on social, political, and economical factors. Side by side, more pragmatic strategies such as well-defined tuberculin test, selective vaccination policy based on tuberculosis incidence in each administrative zone, and early vaccination of high risk groups, should be formulated.

Bacillus Calmette-Guérin (BCG) is the most widely used and as much controversial vaccine in the world. Since its invention in 1921, billions of subjects have already been vaccinated. However, data on its protective efficacy are variable and equivocal. Japan has been conducting universal BCG vaccination in infants and revaccination in school children since 1951 and 1954, respectively.^1^ Annual tuberculosis (TB) incidence decreased from 698 per 100,000 population in 1951 to 31 in 2000,^2^^,^^3^ although big chunk of credit goes to massive socioeconomic development and industrialization after World War II. The protective efficacy of BCG has been heavily questioned after Madras trial in which BCG vaccination failed to show any protection against pulmonary TB.^4^ International controversies surrounding the effectiveness of the BCG,^4^^-^^8^ low incidence of TB among Japanese children and astronomical cost to prevent a case of TB through universal vaccination in infants^9^ have set the stage for a debate whether it is high time to discontinue universal BCG vaccination in Japan. The situation of Japan meets the criteria of International Union against Tuberculosis and Lung Disease (IUATLD)^10^ to discontinue universal BCG vaccination (Table 1). This review is aimed to present current BCG and TB situation in Japan, along with pros and cons on the issue of discontinuation of universal BCG vaccination.

**Table 1.** Japan’s position and IUATLD criteria to discontinue BCG vaccination.

Criteria to discontinue BCG vaccination^9^	Japanese perspectives^1^^-^^3^^,^^11^
The average annual notification rate of sputum smear-positive pulmonary tuberculosis should be 5 cases/100,000 population or less during the previous 3 years.	It was 18.9, 18.8, and 18.4 per 100,000 population during 1994, 1995, and 1996, respectively.
OR	
The average annual notification rate of tuberculous meningitis in children under 5 years of age should be less than one case per 10 million general population over the previous five years.	It was 0.5-0.7 per 10 million general population over the previous five years (1992-1996).
OR	
The average annual risk of tuberculous infection should be 0.1% or less.	It was 0.05% during 2000.

## METHODS

A structured review of published literature, accessible through Medline database and also by manual efforts, was undertaken to obtain relevant data. All abstracts obtained with the key words ‘BCG and Japan’ and ‘TB and Japan’ were scrutinized manually to select relevant studies. Related articles published in Japanese language (both reports and journal articles) were also gathered and necessary data were extracted. Each of the studies and reports was assessed regarding the following data: protective efficacy of BCG vaccine in international and Japanese perspectives, cost-effectiveness of BCG vaccination and revaccination policies in Japan, BCG side effects, BCG injection technique in Japan, discontinuation of BCG vaccination and its effects present TB situation in Japanese perspectives, reasons of non-declining TB incidence in Japan, TB group infection in Japan, and relative situation of Japan compared to other developed countries regarding BCG and TB.

## RESULTS

### Protective efficacy of BCG vaccine

Controversies on BCG vaccine efficacy are widespread and efficacy of BCG vaccination is currently a matter of debate in the international arena.^5^^,^^6^ There are wide ranging variations in the efficacy of BCG vaccine, from detrimental effect to more than 90% protective benefit in prospective trials and case-control studies^7^ with 50% in the meta-analysis of published literatures.^8^ Higher (64%) protective effect against TB-meningitis was observed in a meta-analysis of five studies.^8^ The worst efficacy was found in the Madras trial in which BCG vaccination failed to show any protection against pulmonary TB in either adults or children up to 5 years after vaccination.^4^ Re-evaluation with 15 years interval showed 17% protective efficacy in persons who had been vaccinated as children but no protective efficacy in persons who had been vaccinated after adolescence.^12^ Moreover, BCG efficacy varies widely within and between the countries. There has been a theory that most of the country variations in BCG efficacy are statistically explainable by the latitude i.e., little or no protection in countries at lower latitude.^13^ Atypical mycobacterium which accrue natural protection against TB, are highly prevalent at lower latitudes. BCG thus could do little to protect against TB in those countries at lower latitudes.

Very little literature is available regarding BCG vaccine efficacy in Japan. Nearly 78% of the pediatric TB patients diagnosed in the year of 2000 in Japan had a history of BCG vaccination (Table 2), although it does not tell us that much about vaccine efficacy.^3^ In a hospital based case-control study, 78% efficacy has been estimated in protecting against pulmonary TB.^14^ Unfortunately, these estimates are most likely biased due to extremely high BCG vaccine coverage (95%) in Japan. Actually it has been suggested that in a population with more than 80% vaccine coverage, a case-control study is not a good choice for demonstrating efficacy.^15^ In that case, non-vaccinated individuals are likely to be from lower socioeconomic group with low education profile and least health awareness. These factors are difficult to adjust in a case-control study design unless otherwise it is designed meticulously for prospective data.

**Table 2.** History of BCG vaccination among the pediatric TB patients found during 2000 in Japan.^3^

	Age (year)					
	0-4		5-9		10-14	
Total number of TB	98	(100%)	46	(100%)	72	(100%)
- BCG vaccinated	48	(49%)	36	(78%)	70	(97%)
- Non-vaccinated	50	(51%)	10	(22%)	2	(3%)

BCG revaccination is more controversial than universal vaccination in infants. No single evidence has been generated so far on its effectiveness. Japan has been conducting revaccination policy since 1954 among the first year primary school and first year junior high school children. Strangely enough, however, its effectiveness has never been investigated. Reports from other parts of the world showed BCG revaccination caused slightly decreased childhood and adolescent TB incidence in Hungary^16^ and Poland,^17^ but not in Chile.^18^ A recently concluded controlled trial in Malawi demonstrated that BCG revaccination provided no protection against TB.^19^ Moreover, BCG vaccination was shown harmful in HIV infected individuals where the risk of pulmonary TB actually increased. A study from Finland reported very low or no efficacy in BCG revaccination.^20^ A study from Hong Kong even showed slightly lower TB incidence among non-revaccinated school children than revaccinated individuals.^21^ Furthermore, the World Health Organization has expressed negative attitude toward revaccination in its new recommendation.^22^ On the basis of lack of evidence, Japan discontinued BCG revaccination program from the year 2002.^23^

### BCG injection technique

There is a controversy regarding BCG injection technique in Japan. In a study which compared BCG injection technique employed at a hospital attached to Research Institute of Tuberculosis, in Tokyo, with other institutions, injection procedures employed at the latter institutions were not appropriate.^24^ This technical problem might have further diminished protective effect of BCG in Japan.

### BCG side effects

Literature on BCG side effects in Japan is not rich enough to get the real picture. However, Mori, et al.^25^ investigated the side effects on local lymph node in universal BCG vaccination. According to this report, prevalence of overall lymph node swelling was 1.06%, while lymphadenopathy, suppurative adenitis, and surgical treatment for lymphadenitis after BCG vaccination were estimated to be 0.73%, 0.02%, and 0.006%, respectively. Life-threatening side effects were not investigated systematically, but reported only as anecdotal cases, i.e., severe-combined-immunodeficiency syndrome among infants after universal vaccination.^26^^,^^27^ Literature on BCG side effects published in Japan during 1987-2000 were summarized by Takamatsu, et al. (only moderate and severe side effects other than localized lympadenopathy) (Table 3).^28^ According to this study, nine BCG osteomyelitis, three disseminated BCG infection, and a case of death due to severe immunodeficiency were reported.

**Table 3.** Summary of BCG side effects (other than mild reaction and lymphadenopathy), along with treatment applied and prognosis, reported in Japanese language articles.^28^

Category	Values
Total number of articles reported BCG side effects	108

Total number of cases reported	151
- Purulent lympadenitis	66
- Cutaneous TB	38
- Ulcer, abscess or Koch phenomenon	10
- Osteoarthopathy including osteomyelitis	9
- Eruption including erythema tuberculatum	8
- Multiple lympadenitis, hepatomegaly, splenomegaly	4
- Lupus tuberculosis	3
- Disseminated TB	1
- Leiomyoma	1
- Unknown	11

Treatment applied	
- Anti-TB drug	70
- Surgery	30
- Observation	26
- Unknown	35

Prognosis	
- Recovery	85
- Sequelae	5
- Death	1
- Unknown	60

Very little information is available on the side effects of BCG revaccination in Japan. Although 0.5% of the 1st grade primary school students and 0.9% of the 1st grade junior high school students were reported to have lymphadenopathy found in a snap survey,^29^ few of them visited physicians. Since treatment of BCG side effects is supported by the government, every adverse effect of BCG is notified to the appropriate authority. Accordingly only 19 cases of BCG side effects (lymphadenopathy, local ulceration, and fever) among the children aged 6-15 years were reported in a year.^29^ Two severe disseminated BCG infection among school children were also reported after revaccination (of whom one died).^30^^,^^31^

### Cost-effectiveness of BCG vaccination and revaccination programs

In a recent study, ‘universal BCG vaccination in infants’ and ‘no vaccination’ were compared using data of one year birth cohort (1.2 million) in Japan. Universal BCG vaccination was not found economically justifiable in that study.^9^ Even with flexible vaccine efficacy (40-80%), the cost to prevent one case of TB was US$ 35,950-175,862 (in 1999 US$).^9^ In other words, 2,125-10,399 immunizations are necessary to prevent one case of TB. Using BCG efficacy elicited from meta-analysis (50%), the cost to prevent one case of TB was US$ 124,755 (in 1999 US$).^32^ Again 50% efficacy seems to be an even wishful estimate if we consider the widespread controversies. On the other hand, direct cost to treat an uncomplicated pediatric TB case was only around US $ 8,384.^33^

Revaccination has been conducted in Japan since 1954 among tuberculin negative 1st grade primary school and 1st grade junior high school children. Sixty-six percent of the former group and 38% of the latter group were found tuberculin negative in 1996. In a study of economic evaluation of BCG revaccination it was concluded that US$ 115,147 is necessary to prevent one case of TB through revaccination based on 50% vaccine efficacy in revaccination.^34^ Furthermore, benefit-cost ratio did not reach close to one with even 90% vaccine efficacy and 15 years duration of protection in revaccination.^35^ Strictly speaking, the real picture of vaccine efficacy in revaccination is not encouraging at all. Only very slight or no effectiveness has been observed in extensive literature review.

### Discontinuing universal BCG vaccination and revaccination, and its effects

In Sweden there were an excessive number of BCG osteitis cases as TB incidence fell below 20 per 100,000 population in 1960s and 1970s. To avoid unwanted sequelae of BCG vaccination, universal vaccination was discontinued in 1975.^36^ Following the discontinuation, TB incidence increased temporarily, but it came down and leveled off in a few years.

Universal BCG vaccination was also discontinued in Czech Republic (partly) in 1989.^37^ A retrospective analysis showed that after the discontinuation, the number of new TB cases has increased and vaccine efficacy was calculated as 80% based on these data. However, the total number of TB was so small that BCG vaccination was not cost-effective to be reintroduced.

In Finland, the discontinuation of BCG revaccination had minimum impact on overall incidence of TB among the respective age groups of children with only 2 additional cases reported in non-revaccinated group within 5 years.^20^

### Effects of discontinuation of BCG vaccination in Japan - hypothetical situation

Table 4 shows the hypothetical TB incidence in Japan with and without universal vaccination based on a hypothetical situation that universal BCG vaccination program was discontinued from the year 2000. TB incidence observed in the year 2000 among the 0-14 years aged group^3^ was assumed as the baseline value. TB incidence after discontinuation was calculated with 50% vaccine efficacy using a formula reported in our previous study.^9^ Other hypotheses in this calculation included 10% reduction in TB incidence among children because of greater efficiency of TB prevention, diagnosis and treatment in future in the absence of universal BCG vaccination. On the other hand, TB incidence with universal vaccination was estimated based on present TB incidence with 5% annual discount. Based on these assumptions, TB incidence rate among children would go up without universal vaccination for certain time, followed by the decline again, as observed in other countries.

**Table 4.** Hypothetical TB incidence among 0-14 yrs old population in Japan.^3^^,^^9^

Year	With universal vaccination (per 100,000 population)			Without universal vaccination (per 100,000 population)		

	Age (year)			Age (year)		
	0-4	5-9	10-14	0-4	5-9	10-14
2000	2.3	0.8	1.4	-	-	-
2005	1.8	0.6	1.1	2.7	0.9	1.7
2010	1.4	0.5	0.9	1.7	0.6	1.0

**Hypothetical Calculations:**

Incidence of TB among the 0-14 yrs children, if universal BCG vaccination were discontinued from the year 2000, was calculated from the formula derived from Rahman et al.^9^ with 10% annual discount for prevention practices, better diagnosis and treatment facilities. On the other hand, future TB incidence, if universal BCG is opted to be continued till 2010, was calculated only by discounting at 5% annual rate.

*Example*:

Suppose BCG was discontinued from the year 2000. Incidence of TB among the 0-4 year-old children in the year 2010 would be;

=Incidence of TB among the 0-4 years reported for the year 2000/(1-proportion of children vaccinated * vaccine efficacy) X 1/(1+0.1)^10^

=23/(1-0.95*0.5) X 1/(1.1)^10^

=1.7 per 100,000 population.

In another scenario where BCG would be continued at least up to 2010. Incidence of TB among the 0-4 year-old children in the year 2010 would be;

= Incidence of TB among the 0-4 years reported for tthe year 2000/(1+0.05)^10^

=23/(1.05)^10^

=1.4 per 100,000 population.

### TB incidence in Japan

**Figure 1.**
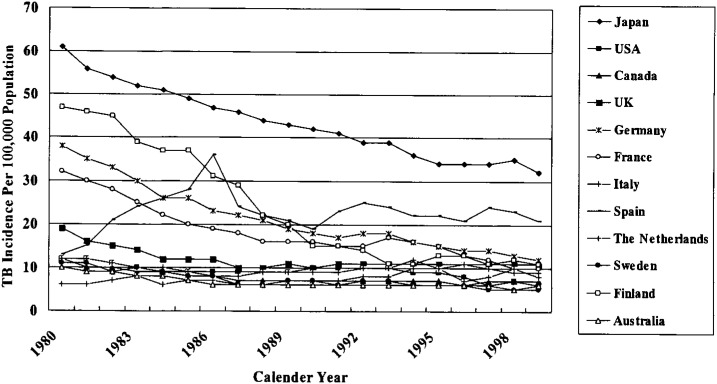
TB incidence in Japan and other developed countries during 1980-1999.^40^

**Figure 2.**
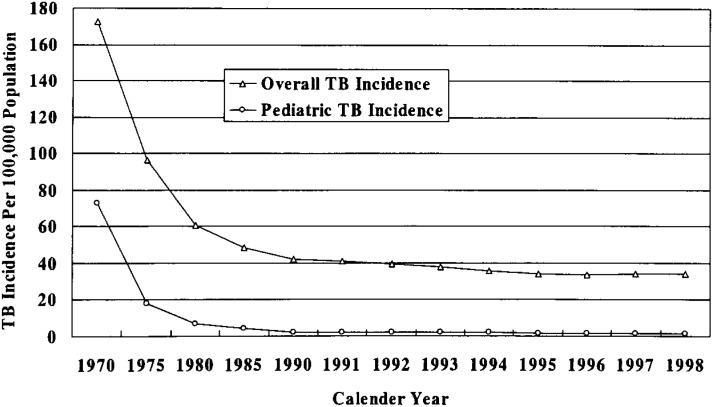
Comparison of overall TB incidence and pediatric TB incidence over time in Japan.^3^

Overall TB incidence in Japan was around 31 per 100,000 population in 2000.^3^ It is not exactly comparable to that of developed countries (Figure 1). It has been declining year-to-year except for 1998 when it went up for the first time in the 38 years. This kind of resurgence did happen in other developed countries including the United States of America and United Kingdom in the 1980s^38^ and the Netherlands in 1995.^39^ Pattern of pediatric and overall TB incidence during 1970-2000 are shown in Figure 2. Pediatric TB incidence in Japan is similar or even lower than other developed countries (for example, 2.1 per 100,000 in Japan vs. 3.1 per 100,000 in the United States of America).^1^

### Trend in decline of TB incidence

**Figure 3.**
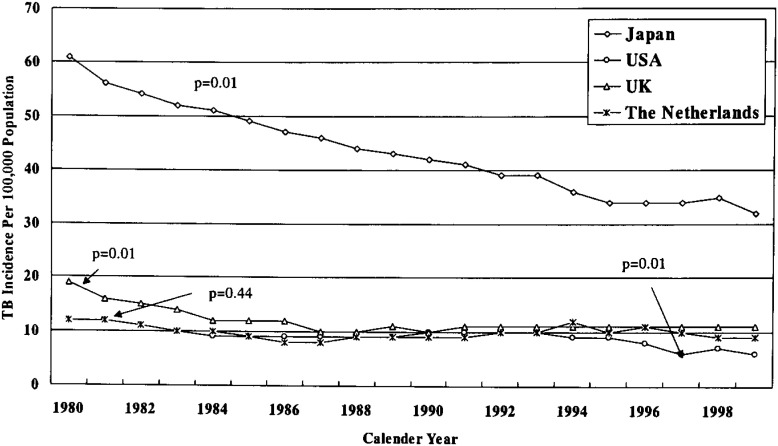
Comparison of TB incidence of Japan with that of other countries (USA and Netherlands) where universal vaccination had never been practiced. Also compared with that of UK where universal vaccination has been practiced in the age of 13-14.^40^ Significant downward trend of TB incidence was observed for Japan, USA, and UK, but not for Netherlands.

TB incidence data^40^ for the past 20 years in Japan and United Kingdom where universal BCG vaccination have been practiced for decades and, also of the United States and the Netherlands where universal vaccination have never been practiced, were analyzed with the use of test for trend (Figure 3). Significant decline in incidence has been observed for Japan (p=0.01), United Kingdom (p=0.01), and the United States (p=0.01), but not in the Netherlands (p=0.44). The reason of non-declining in TB incidence in Netherlands might be due to the fact that TB incidence during 1980s was already very low there (10 per 100,000 in 1980 vs. 8 per 100,000 in 1999).^40^ Thus, the decline in TB incidence in the countries where universal vaccination have never been practiced raises the doubt in actual benefit of BCG vaccination. Again the role of BCG in lowering TB-related mortality is unclear. Countries like USA where universal vaccination has never been performed showed the same rate of decline in mortality due to TB as that of other countries where it has been under universal vaccination program for decades.

### Why TB incidence is not declining in Japan?

In Japan TB incidence declined rapidly until 1982, when it has leveled off. The high TB incidence in Japan is attributable to high TB incidence among the older population groups. For example, TB incidence per 100,000 population among different age groups were: 1.1 - 2.2 among the 0 - 14 year-old age group; 6.8 - 26.5 among 15-49 years; 40.2 - 64.2 among the 50 -69 years; and 108.2 among the 70+ years.^2^ TB epidemics during 1910s in eastern Japan and during 1940s in western Japan, high risk of TB infection (4%) up to World War II,^41^ and lower socioeconomic conditions before the war, were responsible for high infection rate during these years. Thus, majority of the present old groups were infected during their early life in these periods. This high TB incidence among the aged, long life expectancy of Japanese population, expansion of older population in absolute and relative numbers, increase in number of compromised hosts, and sporadic mass outbreak of TB infection among the young subjects, have been contributing to high overall incidence rate in Japan.^42^ Furthermore, non-declining TB incidence can be due to transmission of TB in congregate settings, such as, health care facilities, educational institutions, and shelter for homeless.

### Group TB infection in Japan

Sporadic group infection is now a big problem in Japan. A total of 132 group infections have been reported in Japan in the last 5 years (Table 5). Casualties included 681 clinical TB cases, 2,695 individuals on TB prophylaxis and 1370 cases under close observation with the positive trend over time in different settings.^43^ It is likely that these group infections are contributing to the overall increase in TB incidence in Japan.

**Table 5.** Group TB infection during 1994-1998 in Japan.^43^

	Total number during 1994-1998	Number of clinically significant disease per group TB infection	Number of prophylaxis per group TB infection	Number of close observation per group TB infection
Hospital	32	9.2	18.9	9
Institute	6	7.3	22.3	0
Business Office	39	5.5	8.9	3.8
Community	11	5.3	4.6	1.8
University, College, Technical College	11	1.7	32.7	24.7
High Schools	20	2.2	38.9	6.4
Junior High Schools	11	0.6	30.5	27.3
Primary Schools, Day Care Centers	2	0.5	43.0	107.5
Total	132	5.2	20.4	10.4

Upward trend in the number of group TB infection observed during 1994-1998 (P<0.05)

## DISCUSSION

A silent debate is going on among the TB experts in Japan regarding the necessity of universal BCG vaccination and revaccination in Japan. In an extensive review, it is asserted that universal BCG vaccination in Japan kept TB incidence among children 0-4 yrs old even lower than the United State by inducing TB immunity.^44^ The author also favored BCG revaccination for those who partially or totally lost immunity acquired from primary infection. The author further argued that IUATLD criteria for discontinuation of universal BCG vaccination are conventional. While in another review, another author was for continuing universal BCG vaccination on the ground that it protects children from TB meningitis and military TB.^11^ However, revaccination program was strongly opposed in that review. Based on the fact that there is no evidence of BCG effectiveness in revaccination, government of Japan recently decided to abolish revaccination program.^23^ In the context of continuing debate and changing circumstances a consensus on universal BCG vaccination is also necessary which should be based on social, political and economical factors. Side by side following measures could be helpful to control TB and to determine BCG vaccine efficacy in Japan.

### Selective BCG vaccination based on TB incidence

TB incidence is an important determinant of cost to prevent a case of TB.^9^ There are variations in pediatric TB incidence by region in Japan.^2^ Nineteen of the 47 administrative units (prefectures) had TB incidence about 50% or less than the national average, 11 units about 150 - 200% incidence, and the remaining 17 units almost national average. So the cost to prevent a case of TB would be double for the prefectures where TB incidence is 50% or less of the national average while it would be half for the prefectures where TB incidence is double.^9^ TB incidence has been as low as 20 per 100,000 in some administrative units for the last few years. BCG vaccination policy could be formulated for each prefecture based on incidence data.

### Proposed study design for determining BCG vaccine efficacy determination in Japan

Case-control study is not methodologically appropriate in determining vaccine efficacy when vaccine coverage is beyond the range of 20-80%.^15^ However, it exceeds 95% in Japan.^3^ Randomized controlled trial would not be a good choice due to ethical reasons. The only way remaining is to discontinue universal BCG vaccination in some prefectures where pediatric TB incidence is very low (for example, in Nagano and Yamagata) and to continue close surveillance on the TB incidence.

### TB screening and contact tracing

Tuberculin skin testing is the commonest and cheapest way of tracing TB infection, although interpretation of tuberculin test among BCG vaccinated individuals is difficult. With BCG vaccination, it becomes less clear whether the tuberculin sensitivity reaction is due to vaccination or latent TB infection. There are thus a lot of confusion in deciding who should be investigated further. In addition, implementing preventive therapy in different settings becomes troublesome.^45^ BCG vaccination could thus be playing negative role in TB control.

Currently indiscriminate screening is going on in all over Japan.^11^ Instead, screening program along with contact tracing should be directed to high risk groups i.e., school and university teachers, attendants of day care centers and kindergartens, health care workers, and old aged population. The objective of this strategy would be to prevent and control TB by early detection and treatment of those with active TB.

### Necessary strategies to control TB group infection in Japan

The number of TB group infection has been escalating in every setting in Japan.^43^ It is fueling the TB-related burden by increasing the number of newly infected and diseased population. To control group infection, following strategies could be considered. First, well-defined tuberculin screening policy for high risk groups followed by finding out the extent of group infection by contact tracing is of utmost importance. Policies can be formulated for high risk groups, such as health care workers, school teachers, contacts of TB patients, clinics for older people, and immigrant from high TB prevalent countries. Appropriate guidelines are necessary for management of tuberculin positive individuals. After the discontinuation of universal BCG vaccination and revaccination, tuberculin testing and contact tracing would be an important strategy to control TB in Japan. Second, places where air-conditioning is done by recirculating trapped air (bus, train, buildings including hospital buildings, private chamber of physicians, auditorium etc.) should have new system to exchange air between inside and outside. By this way, floating TB bacillus would not be entrapped inside the closed structure and thereby, likelihood of getting TB infection from active case would go down. Third, in the isolation rooms where TB patients are treated, ventilation systems inside the room should have lower pressure than outside and air should be exhausted directly outdoor. Besides, high-efficiency particulate filtration and ultraviolet germicidal irradiation in high risk areas should be ensured.

The extent of TB transmission from person to person seems to be an important problem in Japan. A study revealed that three-fourth of pediatric TB cases had patients with active TB in their families.^47^ This is the tip of the iceberg of the problem resulting from group infection.

## CONCLUSION

In Japan, there are pros and cons on the issue of universal vaccination program and revaccination. Both groups, for and against, are equally vocal and adamant in showing their logic. Those who oppose, base their logic on two main factors: first, the perceived lack of vaccine efficacy, and second, unacceptable cost-effectiveness of vaccination.^9^^,^^35^ Those who favor it, argue that universal vaccination induces TB immunity and protects against meningeal and military TB while revaccination provides immunity for those who totally or partially lost immunity acquired from the primary vaccination.^11^^,^^44^ In another study, it is reported that discontinuation of universal vaccination is too early for Japan at this moment, and Japan should emphasize on the ‘directly observed therapy’ method, physicians’ knowledge on TB, and accurate BCG injection technique.^23^

This review reveals that a broad consensus considering social, political and economical factors is needed to abolish universal BCG vaccination program in Japan. In the meantime, discontinuing universal BCG vaccination in some low-incidence prefectures, and comparing TB incidence using time-series analysis could be an important strategy to examine BCG effectiveness. After discontinuation of universal vaccination in those areas, BCG vaccination should be considered only for high risk groups.
